# Brain MRI lesions and atrophy are associated with employment status in patients with multiple sclerosis

**DOI:** 10.1007/s00415-015-7853-x

**Published:** 2015-07-24

**Authors:** Shahamat Tauhid, Renxin Chu, Rahul Sasane, Bonnie I. Glanz, Mohit Neema, Jennifer R. Miller, Gloria Kim, James E. Signorovitch, Brian C. Healy, Tanuja Chitnis, Howard L. Weiner, Rohit Bakshi

**Affiliations:** Laboratory for Neuroimaging Research, Department of Neurology, Partners MS Center, Harvard Medical School, Brigham and Women’s Hospital, Boston, MA USA; Novartis Pharmaceuticals, East Hanover, NJ USA; Analysis Group, Inc., Boston, MA USA; Laboratory for Neuroimaging Research, Department of Radiology, Partners MS Center, Harvard Medical School, Brigham and Women’s Hospital, Boston, MA USA; Laboratory for Neuroimaging Research, One Brookline Place, Brookline, MA 02445 USA

**Keywords:** Employment, Productivity, Multiple sclerosis, MRI, Brain atrophy, Brain lesions, Disability

## Abstract

Multiple sclerosis (MS) commonly affects occupational function. We investigated the link between brain MRI and employment status. Patients with MS (*n* = 100) completed a Work Productivity and Activity Impairment (WPAI) (general health version) survey measuring employment status, absenteeism, presenteeism, and overall work and daily activity impairment. Patients “working for pay” were considered employed; “temporarily not working but looking for work,” “not working or looking for work due to age,” and “not working or looking for work due to disability” were considered not employed. Brain MRI T1 hypointense (T1LV) and T2 hyperintense (T2LV) lesion volumes were quantified. To assess lesional destructive capability, we calculated each subject’s ratio of T1LV to T2LV (T1/T2). Normalized brain parenchymal volume (BPV) assessed brain atrophy. The mean (SD) age was 45.5 (9.7) years; disease duration was 12.1 (8.1) years; 75 % were women, 76 % were relapsing-remitting, and 76 % were employed. T1LV, T1/T2, Expanded Disability Status Scale (EDSS) scores, and activity impairment were lower and BPV was higher in the employed vs. not employed group (Wilcoxon tests, *p* < 0.05). Age, disease duration, MS clinical subtype, and T2LV did not differ between groups (*p* > 0.05). In multivariable logistic regression modeling, adjusting for age, sex, and disease duration, higher T1LV predicted a lower chance of employment (*p* < 0.05). Pearson correlations showed that EDSS was associated with activity impairment (*p* < 0.05). Disease duration, age, and MRI measures were not correlated with activity impairment or other WPAI outcomes (*p* > 0.05). We report a link between brain atrophy and lesions, particularly lesions with destructive potential, to MS employment status.

## Introduction

The compromised ability to perform occupational function and unemployment is commonly seen in multiple sclerosis (MS) [1–8]. Impaired work performance is related to a variety of disease manifestations including physical disability, cognitive impairment, psychological factors, pain, and fatigue [1–4, 6–13]. Furthermore, in addition to the financial impact of occupational limitations, such impairment may significantly lower quality of life [2, 14].

MRI of the brain is a valuable tool for the diagnosis and longitudinal monitoring of patients with MS [15]. MRI imaging can depict a variety of effects of the disease process, such as the development of T1 hypointense lesions, T2 hyperintense lesions, and atrophy [15, 16]. A growing body of evidence has linked MRI-defined disease severity to key clinical manifestations such as physical disability, mood disturbances, and cognitive impairment [15–24]. However, to date, the relationship between MRI and occupational function has not been evaluated.

In a recent cross-sectional study, we administered a work productivity scale to patients with MS and identified a role for disability, depression, fatigue, and anxiety in impairment of occupational functions [2]. In the present study, we extended our previous observations in this cohort by examining the available MRI scans to assess the link between brain lesions/atrophy and employment status/productivity.

## Methods

### Subjects and clinical evaluation

As part of the Comprehensive Longitudinal Investigation of Multiple Sclerosis at Brigham and Women’s Hospital (CLIMB) study [25], we previously reported employment-related data on 377 patients with a clinically isolated demyelinating syndrome (CIS) or relapsing-remitting MS (RRMS), based on the administration of the Work Productivity and Activity Impairment (WPAI) questionnaire (general health version) [2].

The WPAI survey, administered during a scheduled clinical visit in the CLIMB study, measured employment status, absenteeism, presenteeism (impairment during work), overall work impairment, and daily activity impairment. Patients who reported “working for pay” were classified as employed (*n* = 76). While those “temporarily not working but looking for work,” “not working or looking for work due to age,” and “not working or looking for work due to disability” were considered not employed (*n* = 16). Patients who were not classified as employed or not employed (*n* = 8) because they were working in home (*n* = 4), volunteering (*n* = 1), or in school (*n* = 3) were excluded from the analysis comparing the employed vs. not employed groups.

During the same clinical visit, patients also underwent a neurologic examination by an MS neurologist to rate physical disability using the Expanded Disability Status Scale (EDSS) [26]. To qualify for this MRI-based sub-study, patients were required to have undergone brain imaging on the assigned CLIMB study MRI scanner using a consistent acquisition protocol that included a 3D high-resolution scan and was performed within 90 days of the clinical visit. One hundred patients were identified, the clinical and demographic characteristics of whom are shown in Table 1.

**Table 1:** Multiple sclerosis patient characteristics

Age, years: Mean ± SD (range)	45.5 ± 9.7 (19.9–64.8)
Female, *n* (%)	75 (75.0)
Race, *n* (%)	
Black or African American	4 (4.0)
South Asian	1 (1.0)
White	94 (94.0)
Unknown	1 (1.0)
Employment status, *n* (%)	
Working for pay	76 (76.0)
Working in home	4 (4.0)
Volunteering	1 (1.0)
In school	3 (3.0)
Temporarily not working but looking for work	3 (3.0)
Not working or looking for work because of age	2 (2.0)
Not working or looking for work because of disability	11 (11.0)
MS disease duration, years: Mean ± SD (range)	12.1 ± 8.1 (1.6–36.6)
MS category, *n* (%)	
Clinically isolated syndrome	6 (6.0)
Relapsing-remitting	76 (76.0)
Secondary progressive	12 (12.0)
Primary progressive	6 (6.0)
Brain parenchymal volume, mL: Mean ± SD (range)	1429.7 ± 70.7 (1216.3–1574.7)
T1 hypointense lesion volume, mL: Mean ± SD (range)	1.3 ± 2.1 (0.0–9.5)
T2 hyperintense lesion volume, mL: Mean ± SD (range)	4.9 ± 6.7 (0.1–28.3)
T1/T2 lesion volume ratio: Mean ± SD (range)	0.3 ± 0.2 (0.0–0.7)
EDSS: Mean ± SD (range)	2.2 ± 1.9 (0.0–7.5)
WPAI:GH, mean ± SD, %	
Activity impairment (*n* = 99)	21.0 ± 26.5
Overall work impairment (*n* = 74)	18.3 ± 25.6
Absenteeism (i.e., percent work time missed, *n* = 74)	6.7 ± 19.7
Presenteeism (i.e., days at work but limited in performing job tasks due to health, *n* = 72)	13.5 ± 20.1

*n* = 100 unless otherwise indicated

*EDSS* expanded disability status scale, *WPAI:GH* work productivity and activity impairment-general health

All subjects gave informed consent. This study was approved by the Partners Health Care ethics committee and was performed in accordance with the ethical standards of the 1964 Declaration of Helsinki and its later amendments.

### MRI acquisition

Brain MRI was performed in all subjects on a 1.5T scanner (GE Signa, Milwaukee, WI). Scan acquisitions covered the whole brain and included an axial T1-weighted spin-echo (TR/TE: 725/20 ms) and dual-echo T2-weighted (TR/TE2/TE1: 3000/80/30 ms) series (voxel size 0.94 × 0.94 × 3 mm) and a sagittal 3D MP-RAGE sequence (TR/TE: 8.6/3.8 ms) with a voxel size of 0.94 × 0.94 × 1.2 mm. T1-weighted spin-echo imaging was repeated 5 min after 0.1 mol/kg intravenous gadolinium (Gd).

### MRI analysis: lesions

Brain T1 hypointense (T1LV) and T2 hyperintense lesion volume (T2LV) were expert-quantified with an edge-finding tool using Jim software (v.5, Xinapse Systems Ltd, West Bergholt, UK, http://www.xinapse.com/). T2 hyperintense lesions were defined as those showing hyperintensity on both the proton density and late echo T2-weighted images. T1 hypointense lesions (“black holes”) were required to show at least partial hyperintensity on the dual-echo images, but no gadolinium-enhancement (to reduce the likelihood of including transient lesions) [15]. To assess a patient’s lesional destructive capability, we calculated the ratio of T1LV to T2LV (T1/T2) for each subject, based on our previous work showing the value of this metric [27–29].

### MRI analysis: atrophy

To assess whole brain atrophy, we measured normalized brain parenchymal volume (BPV) from the 3D MP-RAGE images using the fully automated segmentation-based algorithm, Structural Image Evaluation using Normalization of Atrophy (SIENAX) [30]. Our method has been previously detailed [31]. Briefly, the automated process involved extraction of the brain and CSF volume from the whole-head input data, followed by affine-registration to a standardized space. The volumetric scaling factor was then obtained for normalization by head size. Tissue-type segmentation with partial volume estimation was then conducted to calculate the total volume of brain tissue vs. CSF. Optimization experiments led to our use of the default brain extraction threshold of 0.5 to maintain adequate segmentation in each image set.

### Statistical analysis

Baseline descriptive statistics were summarized, and baseline age, disease duration, EDSS, brain volume measures, and activity impairment were compared between employed vs. not employed patients using the Wilcoxon rank-sum test. Correlations between patient characteristics and WPAI General Health scores were assessed using Pearson’s correlation coefficient for all variables except for EDSS, for which the associations were measured using Spearman’s rho. Logistic regression models were used to explore whether employment status was associated with brain volume and lesion volume. Age, sex, and disease duration were included as covariates in the logistic regression models. All data analyses were performed using SAS release 9.3 (SAS Institute, Inc., Cary, NC).

## Results

**Fig. 1:**
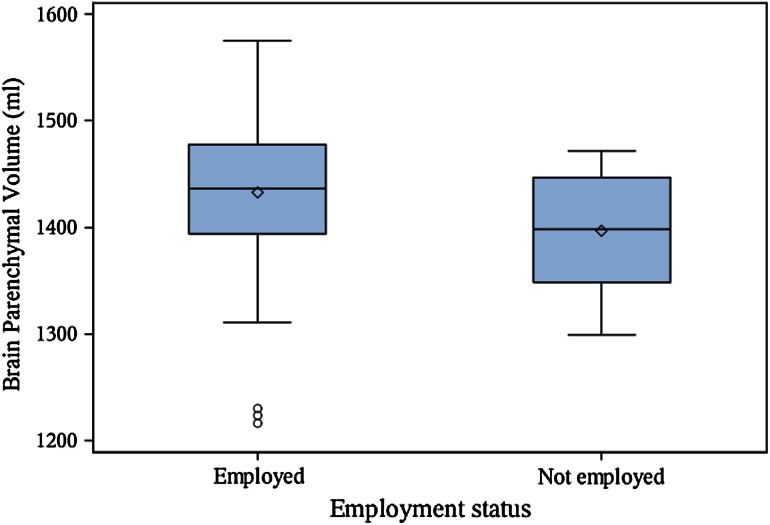
Brain atrophy is associated with MS employment status. *Boxplot* with mean (*diamond*), median, quartiles, 95 % interval whiskers and outliers. Wilcoxon rank-sum test: *p* value = 0.036

**Fig. 2:**
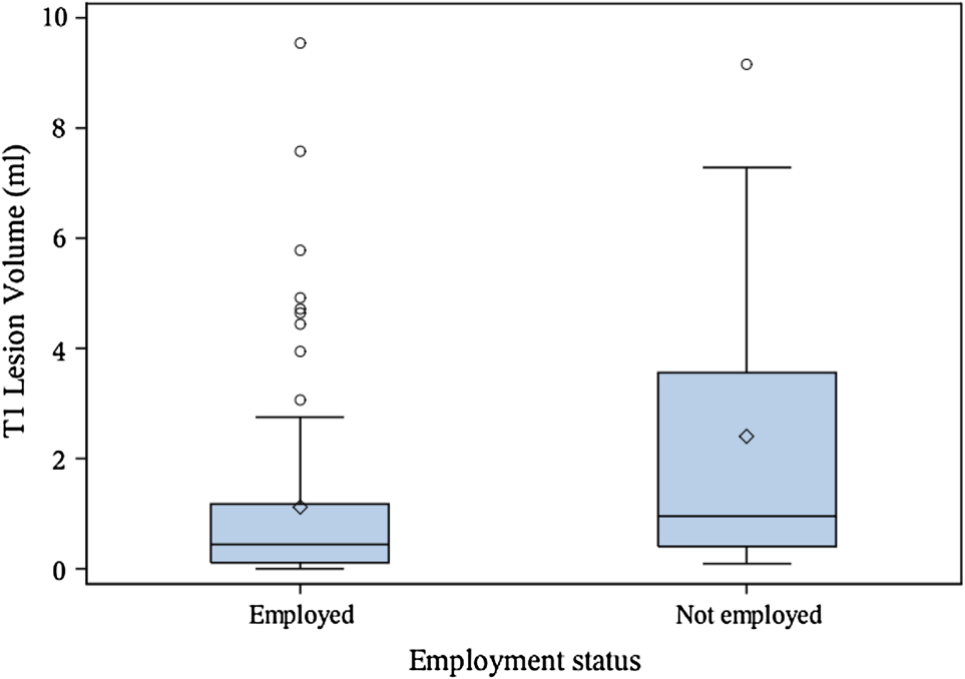
Brain T1 hypointense lesion volume is associated with MS employment status. *Boxplot* with mean (*diamond*), median, quartiles, 95 % interval whiskers and outliers. Wilcoxon rank-sum test: *p* value = 0.014

**Fig. 3:**
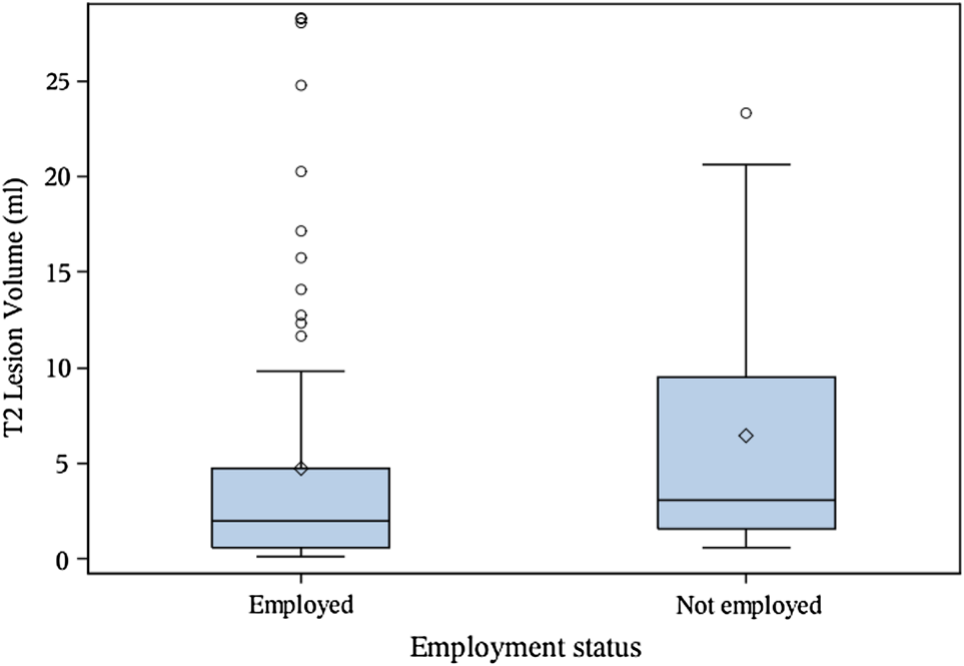
Brain T2 hyperintense lesion volume is not associated with MS employment status. *Boxplot* with mean (*diamond*), median, quartiles, 95 % interval whiskers and outliers. Wilcoxon rank-sum test: *p* value = 0.107

**Fig. 4:**
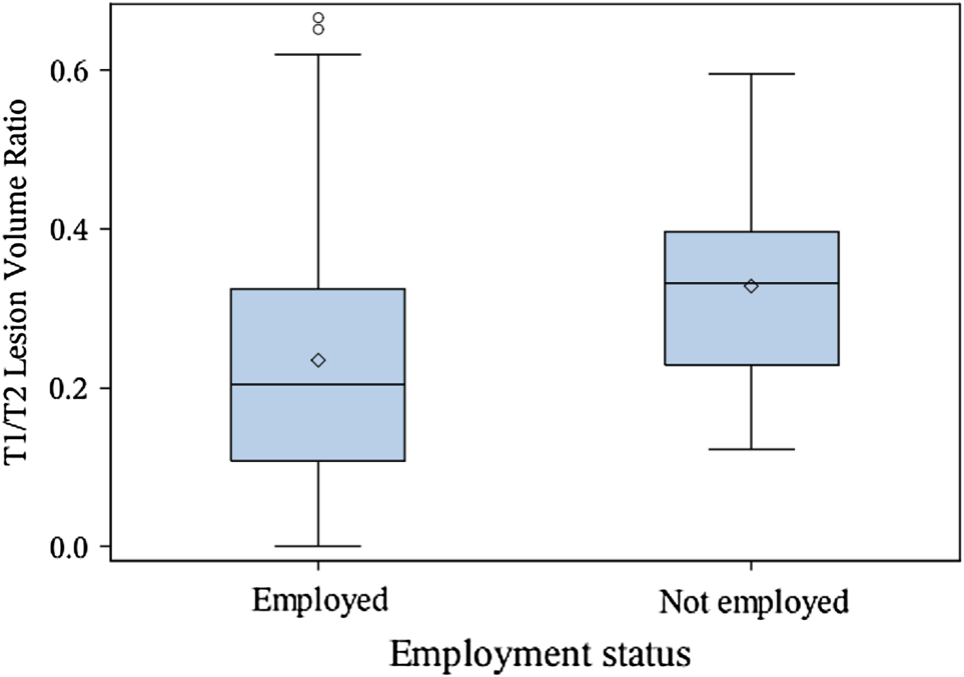
Brain T1/T2 lesion volume ratio is associated with MS employment status. *Boxplot* with mean (*diamond*), median, quartiles, 95 % interval whiskers and outliers. Wilcoxon rank-sum test: *p* value = 0.012

Patient demographic, clinical, and MRI characteristics are shown in Table 1. Among the 100 patients analyzed in the correlation analysis, 74 % had absenteeism and 72 % had presenteeism data. A total of 12 patients had an absenteeism score >0, while 33 patients had a presenteeism score >0. The main results are shown in Tables 2 and 3 and Figs. 1, 2, 3, 4. Seven patients had Gd-enhanced lesions; however, due to power considerations and study design, we did not formally compare patients for the presence or absence of Gd-enhancing lesions in relation to employment in this study. As shown in Table 2 and Figs. 1, 2, and 4, T1LV, T1/T2, Expanded Disability Status Scale (EDSS) scores, and activity impairment were lower and BPV was higher in the employed vs. not employed group (Wilcoxon tests,* p* < 0.05; Wilcoxon rank-sum tests, *p* < 0.05). Age, disease duration, T2LV, and clinical subtype of MS were not significantly different between groups (*p* > 0.05) (Tables 2 and 3, Fig. 3). Spearman’s correlation coefficient showed that EDSS was associated with activity impairment (*p* < 0.05) (Table 4). However, disease duration, age, and MRI variables were not correlated with activity impairment, absenteeism, presenteeism, or overall work impairment (all *p* > 0.05).

**Table 2:** Clinical/MRI multiple sclerosis disease variables in employed vs. not employed patients

	Mean difference employed (*n* = 76) vs. not employed (*n* = 16)	Wilcoxon rank-sum test *p* value^b^
Patient characteristics^a^		
Age^c^	−4.429	0.090
Disease duration	−0.008	0.857
Baseline EDSS	−2.030	<0.001
Brain volume measures^a^		
BPV	3.645	0.036
T1LV	−1.288	0.014
T2LV	−1.739	0.107
T1/T2 LV ratio	−0.093	0.012
Activity Impairment	−27.125	0.002

*EDSS* Expanded Disability Status Scale score, *BPV* brain parenchymal volume, *LV* lesion volume

^a^This analysis was conducted among patients who reported any of the following: “working for pay,” “temporarily not working but looking for work,” not working or looking for work due to age,” and “not working or looking for work due to disability.” Patients who reported “working for pay” were considered employed. BPV, T1, and T2 lesion volumes are measured in mL

^b^ *p* values were estimated using a non-parametric Wilcoxon rank-sum test for two independent samples

^c^When stratified by employment status, the mean age among employed patients was 44.9 (SD 8.6) with a range of 27.1–62.6 years and the mean age among not employed patients was 49.3 (SD 10.5) with a range of 29.8–64.8 years. Note that the regression modeling testing the relationships between MRI and employment status were adjusted for age, sex, and disease duration (see “Results” Section)

**Table 3:** Clinical subtype of multiple sclerosis by employment status

	Employed, *n* (%)	Not employed, *n* (%)
Clinically isolated syndrome	5 (6.6)	1 (6.3)
Relapsing-remitting	60 (78.9)	10 (62.5)
Secondary progressive	6 (7.9)	4 (25.0)
Primary progressive	5 (6.6)	1 (6.3)

There was no significant difference between employed (*n* = 76) and not employed (*n* = 16) groups in terms of the distribution of multiple sclerosis clinical subtype (*p* > 0.05)

**Table 4:** Correlations between clinical/MRI multiple sclerosis disease variables and work productivity

Variables	Patient characteristics (years)		Physical disability	Work Productivity and Activity Impairment-General Health (%)				Brain volume measures			
	Age	Disease duration	EDSS	Activity impairment	Overall work impairment	Absenteeism	Presenteeism	BPV	T1 lesion volume	T2 lesion volume	T1/T2 lesion volume ratio
BPV	−0.370*	−0.199*	−0.219*	−0.150	−0.112	−0.018	−0.132	–	−0.337*	−0.381*	−0.198*
T1 hypointense lesion volume	−0.008	0.147	0.326*	0.157	0.116	0.021	0.138	−0.337*	–	0.869*	0.366*
T2 hyperintense lesion volume	−0.016	0.226*	0.186	0.116	0.062	−0.028	0.090	−0.381*	0.869*	–	0.098
T1/T2 lesion volume ratio	0.173	0.045	0.331*	0.055	0.121	0.147	0.122	−0.198*	0.366*	0.098	–
Baseline EDSS	0.273*	0.286*	–	0.424*	0.195	−0.062	0.211	−0.219*	0.326*	0.186	0.331*
Age at MRI	–	0.374*	0.273*	0.153	−0.015	−0.162	0.123	−0.370*	−0.008	−0.016	0.173
Disease duration	0.374*	–	0.286*	0.197	0.118	−0.039	0.170	−0.199*	0.147	0.226*	0.045

All correlations are Pearson, except for those involving EDSS which are Spearman; Asterisks indicate *p* value <0.05

*BPV* brain parenchymal volume, *EDSS* expanded disability status scale score

### Regression Modeling

To further explore the strength of the relationship between MRI and MS employment status, we developed three logistic regression models (each adjusting for age, sex, and disease duration): (1) T1LV vs. employment status, (2) T1/T2 vs. employment status, and (3) BPV vs. employment status. In the first multivariable logistic regression model, we found a statistically significant reduction (*p* = 0.047) in the odds of being employed vs. unemployed by 22.4 % for each 1 mL increase in T1LV. We did not observe a significant association between T1/T2 (*p* = 0.144) or BPV (*p* = 0.191) and employment status in the second and third multivariable models.

## Discussion

The major finding in this study is the increased disease severity as shown by MRI-defined cerebral lesions and atrophy in MS patients who are not employed vs. those who are employed. Among lesion measures, the destructive potential as assessed by the overall T1 hypointense burden and the proportion of T2 hyperintense lesions showing T1 hypointensity were most strongly related to the employment status. In addition, normalized whole brain volume was higher in employed vs. not employed patients, suggesting that brain atrophy was also a factor linked to not employed status.

These findings are perhaps not surprising given that a range of neurological and neuropsychological dysfunction in MS, such as physical disability, cognitive impairment, and mood disturbances, have been linked to both impaired work performance [1–4, 6–13] and brain structural damage as defined on MRI scans by lesions and atrophy [22, 32]. Among MRI lesion measures, T1 hypointense lesions have shown both more specificity for destructive irreversible damage [33] and better correlations with mental and physical impairment [24, 34] than T2 hyperintense lesions. These previous studies are in line with our results that T1 hypointense lesions are also more important for employment status than T2 hyperintense lesions.

The second major finding was that none of the MRI variables were associated with activity impairment in the whole cohort or any work productivity measures in the employed patients, including overall work impairment, absenteeism, or presenteeism. These aspects of impairment were not common in our sample. Thus, the study may have been underpowered to detect such associations due to the restricted range of activity impairment. Secondly, the latter three measures of impairment are only relevant to employed patients and thus may not have provided sufficient sensitivity. Furthermore, these self-reported measures may not have been reliable. We did not assess any objective measures of productivity while working. Finally, the presence of cognitive reserve [35], which was not assessed in our study, may have provided adaptive ability for patients to maintain function, despite the accumulation of disease-related structural brain changes.

A third finding was the strong relationship between physical disability, assessed by EDSS score, and both employment status and activity impairment. Such a relationship has been long known in MS. However, it was striking in our data that EDSS score showed a closer link than the MRI variables to employment and activity impairment. This important role for disability was seen from two perspectives. First, the differences between employed and not employed groups were robust for EDSS (*p* < 0.001), while less robust for MRI brain atrophy and T1 hypointense lesion variables (*p* < 0.05). Second, EDSS score, but not MRI variables, showed a significant correlation with activity impairment. One possible explanation for this divergence is the heavy contribution of spinal cord involvement to the EDSS score [26], whereas only brain MRI measures were applied in this study. It is likely that spinal cord involvement, a major contributor to limb and ambulatory disability [29, 36–41], is a key contributor to vocational skills, and is poorly reflected in the level of MRI-defined brain lesions or atrophy in patients with MS [36].

Our cohort was dominated by mildly affected patients with relapsing forms of MS. Given that only 18 % of our patients had progressive forms of MS, further studies are required to assess the link between brain MRI findings and employment in advanced forms of the disease. Sample size should also be taken into account as the not employed group comprised 16 patients. We are now planning to study larger cohorts. Additional work is necessary to establish whether the relationships between MRI and employment are independent from the effects of cognitive impairment and fatigue. Because of sample size and the restricted range of employment impairment, there may have been limited power to detect the full extent of MRI relationships. It would be of interest for future studies to test whether other aspects of MRI-defined involvement in MS, such as spinal cord [36–41], cortical [42, 43], and diffuse cerebral damage [44–46], are related to productivity and whether MRI findings predict longitudinal change of employment status. Destructive effects of lesions may be particularly prominent using ultra-high-field strength MRI [47]. In summary, our study demonstrates a clear relationship between brain MRI and employment status in MS.
